# J protein mutations and resulting proteostasis collapse

**DOI:** 10.3389/fncel.2014.00191

**Published:** 2014-07-08

**Authors:** Carolina Koutras, Janice E. A. Braun

**Affiliations:** Department of Physiology and Pharmacology, Hotchkiss Brain Institute, Cumming School of Medicine, University of CalgaryCalgary, AB, Canada

**Keywords:** sacsin, Tim14, Rme-8, auxilin, CSPα, HSJ1, Mrj, Hsp40

## Abstract

Despite a century of intensive investigation the effective treatment of protein aggregation diseases remains elusive. Ordinarily, molecular chaperones ensure that proteins maintain their functional conformation. The appearance of misfolded proteins that aggregate implies the collapse of the cellular chaperone quality control network. That said, the cellular chaperone network is extensive and functional information regarding the detailed action of specific chaperones is not yet available. J proteins (DnaJ/Hsp40) are a family of chaperone cofactors that harness Hsc70 (heat shock cognate protein of 70 kDa) for diverse conformational cellular tasks and, as such, represent novel clinically relevant targets for diseases resulting from the disruption of proteostasis. Here we review incisive reports identifying mutations in individual J protein chaperones and the proteostasis collapse that ensues.

## J Proteins: personal travel guides

**Figure 1:**
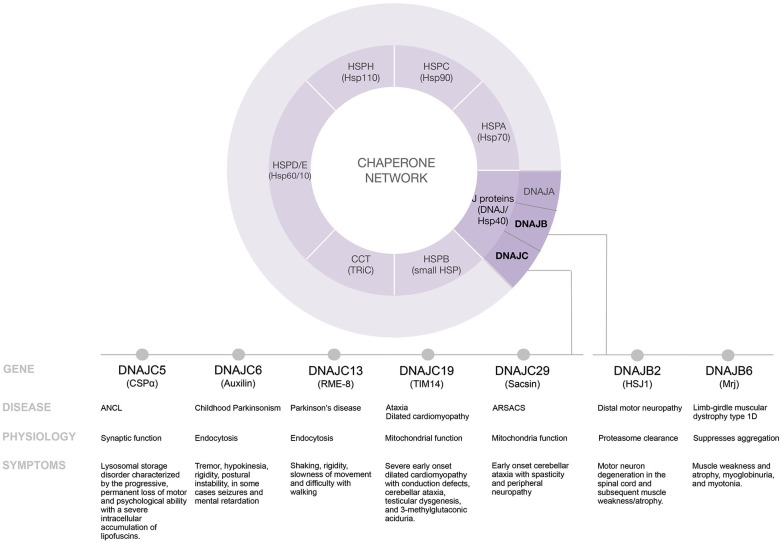
**Disease modifying pathways of select J proteins**.

In neurons, there are significant demands on cellular folding events. Dynamic protein complexes are central to synaptic transmission, a process that occurs with speed, precision and plasticity. Most proteins can exist in more than one conformation and many proteins must change conformation and activity regularly. Rigorous quality control mechanisms operate at the synapse to provide defense against the detrimental effects of functionally impaired proteins and protein complexes. The balance between protecting protein functional integrity and preventing accumulation of misfolded proteins is accomplished by a network of chaperone families including: J proteins (DnaJ/Hsp40), HspA (Hsp70), HspB (small Hsp) HspC (Hsp90), HspD/E (Hsp60/Hsp10), HspH (Hsp110), CCT (TRiC) and numerous regulatory factors (Figure 1). Operating like a switch, J proteins control Hsc70 (70–kDa heat shock cognate protein) by directing and activating Hsc70’s ATPase activity for conformational and refolding work (Kampinga and Craig, 2010; Kakkar et al., 2012). Sequence analysis has highlighted the diversity of the J protein family but has not provided much clarity into the functionality of specific J proteins. Each J protein has a 70 amino acid signature region comprised of four helices, called a J domain. Outside of the J domain, J proteins are structurally divergent, likely the basis for their distinct functional properties. Originally named Hsp40 or DnaJ after the founding members of the family (Georgopoulos et al., 1980; Ohtsuka et al., 1990), we now know that there are 49 J proteins in humans that range in molecular weight from 18–520 kDa and that while some J proteins are induced by heat most members of the J protein family are constitutively expressed (Zhao et al., 2008; Kakkar et al., 2012). The recognition that Hsc70 serves as a central hub and J proteins are the switches for diverse proteostasis events has fueled investigations into understanding the specific role of J proteins.

## Age-associated neurodegenerative disorders

How do folding processes go awry in age? To counteract aging and disease, neurons must routinely regulate protein machinery within tight limits. The quality control machinery constantly protects against the misfolding and aggregation of proteins that is inherent to continuous protein synthesis, generation of folding intermediates and molecular crowding of neural proteins. Neurons which don’t undergo cell division are vulnerable to infectious misfolded aggregated proteins that spread (e.g., prion diseases), mutations that produce toxic proteins characteristic of late onset of neurodegenerative disorders such as Alzheimer’s disease, Parkinson’s disease, Huntington’s disease, and Amyotrophic Lateral Sclerosis (ALS) as well as mutations that generate ineffective chaperones. In fact, at the cellular level, pathology may start long before the onset of clinical symptoms associated with failure of the chaperone machinery to meet the demands of misfolded proteins. While it is widely accepted that J proteins maintain and restore protein balance several questions remain regarding the nature of changes to the chaperone network in age related neurodegenerative diseases (Soti and Csermely, 2002; Muchowski and Wacker, 2005; Kim et al., 2013; Labbadia and Morimoto, 2014).

## Defining a trajectory to understand the function of specific J proteins

The chaperone network is elaborate and elucidating the function of individual J proteins is challenging. Chaperonopathies are a collection of diseases caused by genetically inherited mutations in chaperones. Our growing appreciation that diverse diseases in humans result from select J protein mutations provides new roads to explore the mechanisms of action of individual J proteins. To date, mutations in seven distinct J proteins; DNAJB2, DNAJB6, DNAJC5, DNAJC6, DNAJC13, DNAJC19 and DNAJC29 have been linked to distinct diseases in humans (Table 1). Here we review the information that these recently identified mutations provide regarding individual J protein function and the collapse of proteostasis. Understanding the mechanisms of specific J proteins is key to understanding the proteostasis machinery and will be critically important for J protein-based drug development.

**Table 1:** **J proteins mutations and resulting proteostasis consequences in human diseases**.

Human Disease	Chaperone	Mutation	References
Autosomal-recessive distal hereditary **motor neuropathy**	**DNAJB2 (HSJ1)** 32 kda (cytosolic) HSJ1a 38 kda (endoplasmic reticulum) HSJ1b	Splice mutation	(Blumen et al., 2012)
Autosomal-dominant limb-grid **muscular dystrophy**	**DNAJB6 (Mrj)** 26 kda	Phe93Leu Phe89Ile Pro96Arg Splice mutation	(Harms et al., 2012; Sarparanta et al., 2012; Sato et al., 2013; Suarez-Cedeno et al., 2014)
Adult onset autosomal-dominant, **neuronal ceroid lipofusinosis** (ANCL)	**DNAJC5 (CSPα)** 35 kda	Leu115Arg Leu116del	(Benitez et al., 2011; Nosková et al., 2011; Velinov et al., 2012)
Autosomal-recessive juvenile **parkinsonism**	**DNAJC6 (Auxilin)100 kda**	80 kb deletion *(Exons 5–19)* Gln734X Splice mutation	(Edvardson et al., 2012; Vauthier et al., 2012; Köroğlu et al., 2013)
Adult onset autosomal-dominant **Parkinson’s disease**	**DNAJC13 (RME-8)** 220 kda	Asn855Ser	(Vilariño-Güell et al., 2014)
Autosomal-recessive dilated cardiomyopathy (DCMA) and cerebellar **ataxia**	**DNAJC19 (TIM14)** 18 kda	Single nucleotide deletion Frameshift mutation	(Davey et al., 2006; Ojala et al., 2012)
Autosomal-recessive spastic **ataxia** of Charlevoix-Saguenay (ARSACS)	**DNAJC29 (Sacsin)** 521 kda	Asp168Tyr Leu308Phe Leu556Pro (>100 mutations)	(Bouchard et al., 1978; Engert et al., 2000; Thiffault et al., 2013)

## DNAJB2 (HSJ1)

Mutation of DNAJB2 (HSJ1), a 32/38 kDa J protein, causes recessive distal hereditary motor neuropathy (Blumen et al., 2012). Originally identified in Morocco, individuals show a distal motor weakness, hypotonia, atrophy and paralysis of the lower limbs in young adulthood (age 20) due to progressive degeneration of motor neurons in the spinal cord.

HSJ1 was first identified via expression cloning with antiserum prepared against Alzheimer’s disease brain extracts enriched in helical filaments (Cheetham et al., 1992). It is preferentially expressed in neurons where it is proposed to promote proteasome degradation of proteins tagged with polyubiquitin (Cheetham et al., 1992; Chapple and Cheetham, 2003; Westhoff et al., 2005; Howarth et al., 2007). Alternative splicing produces two isoforms. HSJ1a is a cytosolic 32 kDa form and HSJ1b is a 38 kDa form that is anchored to the cytosolic face of the endoplasmic reticulum via C terminal geranylgeranylation. In addition to its J domain, HSJ1 contains two unique ubiquitin interaction domains that bind ubiquitin to prevent aggregation and direct ubiquitinated/misfolded proteins to the proteasome. *Which misfolded proteins are targeted by HSJ1?* In cellular models HSJ1a suppresses the aggregation of polyglutamine expanded proteins and mutant superoxide dismutatse1 (SOD1; Chapple et al., 2004; Westhoff et al., 2005; Gao et al., 2011; Blumen et al., 2012). Transgene upregulation of HSJ1a reduces brain huntingtin aggregation in the mouse R6/2 model of Huntington’s disease (Labbadia et al., 2012) and mutant SOD1 aggregation in the mouse SOD1^G93A^ model of ALS (Novoselov et al., 2013). Key questions arise from these findings. While transgenic expression of HSJ1 alone reduces aggregation, it does not fully reverse disease progression and correct life span. Whether reinforcing other J proteins along with HSJ1 will completely circumvent the cascade of degeneration remains an open question. Further, while the splice mutation that give rise to distal motor neuropathy in humans decreases the availability of both HSJ1a and HSJ1b which are broadly expressed in neurons, the mutation causes selective loss of motor neuron function. Obviously, whether HSJ1 has a motor neuron specific or a general neuronal function will be the focus of future investigation.

## DNAJB6 (Mrj)

Mutations in DnaJB6 (Mrj; mammalian relative of DnaJ), a 26/36 kDa J protein cause autosomal dominant limb-girdle muscular dystrophy type 1 D (Harms et al., 2012; Sarparanta et al., 2012; Sato et al., 2013; Suarez-Cedeno et al., 2014). Four mutations F96L, F96I, F89I and F93L results in adult or child onset (age 14–68 yrs) limb-girdle muscular dystrophy type 1D which is clinically characterized by elevated serum creatine kinase levels, progressive muscle weakness mainly in the legs. Limb-girdle muscular dystrophies are a heterogeneous group of inherited disorders caused by a number of dominant or recessive mutations that cause myofibrillar myopathy and the DNAJB6 mutations are specifically distinguished by characteristic protein aggregates and autophagic vacuoles (Mitsuhashi and Kang, 2012).

Mrj is a ubiquitous J protein with Hsc70-dependent and -independent activities that is most highly expressed in brain (Chuang et al., 2002; Hageman et al., 2010). Further, expression of Mrj is increased in astrocytes from Parkinson’s disease patients, where it is found to be a component of Lewy bodies (Durrenberger et al., 2009). Alternative splicing produces two isoforms (Hanai and Mashima, 2003). DnaJB6a is 36 kDa and localizes to the nucleus, whereas DnaJB6b is 26 kDa and cytosolic but translocates to the nucleus in response to cellular stress (Andrews et al., 2012). *What are Mrj’s targets?* Mrj suppresses aggregation and toxicity of several aggregation-prone proteins including: huntingtin, α-synuclein and parkin (Chuang et al., 2002; Durrenberger et al., 2009; Hageman et al., 2010; Kampinga and Craig, 2010; Rose et al., 2011). Possible “client proteins” in addition to disease-causing proteins include: keratin-intermediate filaments (Watson et al., 2007), histone deacetylase (HDAC; Hageman et al., 2010) and the transcription factor, NFATc3 (Dai et al., 2005). Mrj KO is embryonic lethal in mice due to a failure of chorioallantoic attachment in placental development (Hunter et al., 1999). All human mutations causing myopathy are found in a glycine-phenylaline linker region that follows the amino terminal J domain and result in a reduction in the inhibition of protein aggregation (Sarparanta et al., 2012). Furthermore, low expression of Mrj has been linked to breast cancer (Andrews et al., 2012). It remains an open question why mutations in neuronal-enriched Mrj cause myopathy and low levels are linked to cancer rather than neurodegeneration. Further investigation is required to understand these somewhat disparate pieces and develop a clear picture of the neuroprotective actions of Mrj. It is noteworthy that in the wobbler mouse, an ALS model of progressive motor neuron degeneration, DnaJB3, which shares 90% identity with Mrj(DnaJB6), shows reduced expression in the spinal cord prior to loss of motor neurons (Boillée et al., 2002). Overlap in Mrj and DnaJB3 expression as well as possible overlap in DnaJB6/DnaJB3 client proteins remains to be determined.

## DNAJC5 (CSPα)

The DnaJC5 gene encodes CSPα, a 35 kDa secretory vesicle protein. Mutations in DnaJC5, cause adult onset, neuronal ceroid lipofusinosis (ANCL), a neurodegenerative disorder characterized by lysosomal accumulation of autofluorescent oxidized lipid and protein waste, called lipofuscin (Benitez et al., 2011; Nosková et al., 2011; Velinov et al., 2012). Deletion of leucine 116 (L116Δ), or mutation of residue 115 from leucine to arginine (L115R) results in ANCL which is clinically characterized by anxiety, depression, speech difficulties, ataxia, myoclonus, involuntary movements, progressive seizures and dementia. Neuronal ceroid lipofuscinoses (NCLs) are a heterogeneous group of inherited lysosomal storage disorders caused by mutations that lead to accumulation of lipofuscin and loss of neurons (Anderson et al., 2013). ANCL is unique among NCLs in that it is adult onset and the only NCL mutations inherited in an autosomal dominant manner.

*What is the physiological function of CSPα?* CSPα protects against synapse loss, however the precise molecular mechanism underlying neuroprotection is not yet known. In addition to its N terminal J domain, CSPα bears a hydrophobic region followed by the distinctive cysteine string region after which the protein is named. Most of the cysteines are palmitoylated, which anchors CSPα to the synaptic vesicle membrane. CSPα is located on synaptic vesicles (Mastrogiacomo et al., 1994), exocrine vesicles (Braun and Scheller, 1995), and endocrine vesicles (Chamberlain et al., 1996; Brown et al., 1998). At birth, CSPα KO mice appear normal and around postnatal day 20 develop progressive motor deficits and CNS degeneration, followed by early lethality (Fernández-Chacón et al., 2004). The synapse loss in CSPα null mice is activity-dependent and synapses that fire frequently are lost first (Schmitz et al., 2006; Garcia-Junco-Clemente et al., 2010). In *Drosophila*, CSPα KOs that survive to adulthood are characterized by uncoordinated movements, shaking, and temperature-sensitive paralysis (Zinsmaier et al., 1994). *What are CSPα’s targets?* Presynaptic targets that demonstrate changes in protein levels early-on in the cascade of neurodegeneration include SNAP25 (Sharma et al., 2011, 2012), dynamin1 (Zhang et al., 2012) and BK channels (Kyle et al., 2013; Ahrendt et al., 2014). Other promising targets include; voltage dependent Ca^2+^ channels, heterotrimeric G proteins, syntaxin and synaptotagmin (Donnelier and Braun, 2014). Which client proteins are critical for triggering the cascade of events leading to degeneration and which changes are downstream of the primary event remains an open question. In addition to CSPα, CSPβ and CSPγ have been identified in the mammalian genomes (Fernández-Chacón et al., 2004). While brain expresses only CSPα, all CSP isoforms are expressed in testis, whereas the cochlea expresses CSPα and CSPβ (Chandra et al., 2005). In CSPα KO mice, the redundancy of CSPβ in the ribbon synapses protects against neurodegeneration in cochlea, however other J proteins do not protect against the absence of CSPα. Surprisingly, in mice, transgenic expression of α-synuclein abolishes neurodegeneration caused by deletion of CSPα but its protective mechanism has not yet been fully elucidated (Chandra et al., 2005). The L116Δ and L115R mutations that result in ANCL cause CSPα to mislocalize implying both a partial loss of function (at the synaptic vesicle) and a partial gain of function (at the intracellular site of mislocalization). Although progress has been made, mislocalization is not sufficient to explain lysosome dysfunction. There are still gaps in our understanding of the molecular mechanisms underlying CSPα’s neuroprotection with regards to synaptic vesicle release and recycling (Rozas et al., 2012; Sharma et al., 2012).

## DNAJC6 (Auxilin)

Mutations in DnaJC6 (auxilin), a 100 kDa nerve-specific J protein cause recessive juvenile parkinsonism characterized by early onset (age 3–18) tremor at rest, bradykinesia, rigidity and postural instability (Edvardson et al., 2012; Vauthier et al., 2012; Köroğlu et al., 2013). The severity and age of onset depends on the extent of the deletion or reduction in expression of auxilin and in some cases includes mental retardation, epilepsy and lack of responsiveness to L-Dopa.

Auxilin is one of the best studied J proteins and its role in uncoating of clathrin from clathrin-coated vesicles is well established (Ungewickell et al., 1995; Eisenberg and Greene, 2007). Neurotransmission requires a rapid continuous recycling of synaptic vesicles. Following fusion, clathrin assembles into a lattice on the presynaptic plasma membrane and deforms plasma membrane into a clathrin coated vesicle that is severed from the membrane by dynamin. After internalization auxilin binds clathrin and Hsc70 and in a J domain-dependent process uncoats clathrin to replenish the pool of synaptic vesicles (Morgan et al., 2001). Unpolymerized clathrin remains in association with Hsc70 until released by nucleotide exchange factors for additional rounds of endocytosis (Morgan et al., 2013). The clathrin binding motif and J domain are located at the C terminus of auxilin and therefore C terminal truncations are expected to render auxilin incapable of uncoating clathrin thereby impairing synaptic transmission (Morgan et al., 2001). Extending on these studies, impaired dopamine receptor recycling may explain the lack of L-Dopa responsiveness observed in some auxilin mutations. Auxilin KO mice have a high rate of postnatal mortality and surviving pups have a low body weight and show recycling and endocytosis defects (Yim et al., 2010). There is a high homology between neural-specific auxilin (DnaJC6) and ubiquitous GAK (cyclin G associated kinase; DnaJC26) with primary differences in the N terminal domain and GAK is thought to partially compensate for auxilin deletion. Interestingly, genome wide association studies have linked DnaJC26 (GAK) to Parkinson’s disease susceptibility (Li et al., 2012).

## DNAJC13 (RME-8 receptor mediated endocytosis)

Mutation of DnaJC13 (RME-8 receptor mediated endocytosis 8) a 220 kDa J protein (Asn855Ser) cause adult onset autosomal-dominant Parkinson disease (Vilariño-Güell et al., 2014). Originally discovered in Saskatchewan, onset of disease is between 59 and 85 years and is characterized by slowly progressive tremor, rigidity, bradykinesia and roughly half of the Lewy body inclusions are immunoreactive for DnaJC13. Interestingly, DanJC13 mutation (Ala2057Ser) has also been linked to Tourette syndrome/chronic tic phenotype in patients of European ancestry (Sundaram et al., 2011).

RME-8 was first identified in a screen for endocytotic defects in *C. elegans* (Zhang et al., 2001) and was subsequently shown to have a role in endocytosis in *Drosophila* (Chang et al., 2004) and endosomal function in mammals (Girard et al., 2005). Surprisingly, Rme-8 which bears a central J domain, is widely expressed but not especially abundant in brain (Girard et al., 2005). Rme-8 interacts with retromer (Popoff et al., 2009) and WASH complexes (Freeman et al., 2014) and loss of RME-8 disrupts cation independent-mannose-6-phosphate receptor and epidermal growth factor receptor trafficking (Girard and McPherson, 2008; Popoff et al., 2009). There are numerous gaps in our understanding of how disruption of normal Rme-8 function in endosomal recyclying causes Parkinson disease.

## DNAJC19 (TIM14 mitochondrial import inner membrane translocase subunit 14)

Mutation of DnaJC19 (Tim14 or Pam 18), an 18 Kda J protein, cause early-childhood-onset (before 3 years) recessive dilated cardiomyopathy and cerebellar ataxia (Davey et al., 2006; Sparkes et al., 2007; Ojala et al., 2012). Originally identified in Alberta Dariusleut Hutterites, DnaJC19 mutations are clinically characterized by raised levels of 3-methylglutaconic acid, a readout of mitochondrial distress, dilated cardiomyopathy, prolongation of the QT interval in the electrocardiogram and cerebellar ataxia (Davey et al., 2006).

DnaJC19 is a transmembrane component of the TIM23 mitochondria import machinery that delivers nuclear encoded proteins to the mitochondrial matrix in an ATP dependent manner (Sinha et al., 2014). DnaJC19 activates mortalin, the mtHsp70 ATPase, and activation is counteracted by MAGMAS, a protein that contains a C-terminal J-like domain that lacks the HPD motif required to recruit and activate the Hsc70 ATPase activity. There are multiple mitochondrial J proteins and our understanding of the differences in function and specificity remains limited. Clearly, DNAJC19 and another mitochondrial J protein, DNAJC15, are not redundant as DnaJC15 does not rescue DnaJC19 mutations that lead to dilated cardiomyopathy and cerebellar ataxia. This underscores the importance of identifying the diverse disease modifying pathways of mitochondrial J proteins.

## DNAJC29 (Sacsin)

Mutations in DnaJC29 (sacsin), a 521 kDa J protein, cause early-childhood-onset (age 1–2 years), recessive spastic ataxia of Charlevoix-Saguenay (ARSACS; Bouchard et al., 1978). Originally discovered in Quebec in 1978 (Bouchard et al., 1978), we now know that >100 different mutations in DnaJC29 mutation exist worldwide (Engert et al., 2000; Thiffault et al., 2013). ARSACS is clinically characterized by unsteady gait, spasticity, ataxia, muscle atrophy, due to progressive cerebellar atrophy and peripheral neuropathy. Quebec patients never walk properly and are wheelchair-bound on average by 41 years with life expectancy approximately 51 years. Outside of Quebec, mild ARSACS with onset delayed until later childhood and early 20’s or severe ARSACS with mental retardation is also found (Baets et al., 2010; Thiffault et al., 2013). Deposits of lysosome derived oxidized lipid and protein waste, called lipofuscin, are found in cerebellar cortical neurons or skin (Stevens et al., 2013), somewhat reminiscent of DnaJC5 mutations that cause ANCL.

Sascin is the largest J protein, bearing C termini J domain, an N-terminal ubiquitin-like domain and a higher eukaryotic and prokaryotic nucleotide binding domain (HEPN; Parfitt et al., 2009; Kozlov et al., 2011). Sacsin is predominantly cytosolic with a mitochondria component, it interacts with dynamin-related protein 1, a GTPase required for mitochondrial fission (Kozlov et al., 2011; Girard et al., 2012; Thiffault et al., 2013). Knockdown of sacsin in SH-SY5Y cells results in an overly interconnected and functionally impaired mitochondrial network with mitochondria accumulating in the soma and proximal dendrites (Girard et al., 2012). Sacsin KO mice display age-dependent neurodegeneration of cerebellar Purkinje cells most likely due to defects in mitochondrial proteostasis (Girard et al., 2012). Much work will be needed to untangle the complexities of sacsin mutations and ensuing neurodegeneration.

## Future prospects

Independent threads have recently begun to provide insight into how select J proteins in the protein quality machinery monitor and adjust proteotoxic imbalances in order to maintain neural function. Many knowledge gaps in our understanding of the functionality of specific J proteins exist. It remains to be seen whether identification of further human mutations will yield insights into the biological roles of J proteins. Ultimately, systematic analysis of the mechanisms by which J proteins facilitate proteostasis will enable us to develop novel therapeutic agents and re-purpose drugs currently used for other indications.

## Conflict of interest statement

The authors declare that the research was conducted in the absence of any commercial or financial relationships that could be construed as a potential conflict of interest.
